# Outcomes of refractory and early‐relapsed large B‐cell lymphoma patients after first‐line treatment in the prospective Real‐world dAta in LYmphoma patients and Survival in Adults (REALYSA) cohort in the pre‐CAR T era

**DOI:** 10.1111/bjh.70812

**Published:** 2026-08-30

**Authors:** Stéphanie Guidez, Aurélien Belot, Fanny Cherblanc, Bastien Lesné, Sylvain Carras, Catherine Thieblemont, Fontanet Bijou, Cédric Rossi, Luc Mathieu Fornecker, Jehan Dupuis, Krimo Bouabdallah, Ludovic Fouillet, Adrien Chauchet, Benoit Tessoulin, Loic Ysebaert, Alice Pauc, Pascale Cony‐Makhoul, Remy Gressin, Gaelle Laboure, Antoine Bonnet, Sandy Amorim, Christophe Fruchart, Roch Houot, Gian Matteo Pica, Carole Soussain, Sophie Dennetiere, Cécile Leyronnas, Pauline Lionne‐Huyghe, Hervé Ghesquières, Pierre Sesques

**Affiliations:** ^1^ Hematology Department University Hospital of Poitiers Poitiers France; ^2^ Biostatistics Department LYSARC, The Lymphoma Study Academic Research Organization Lyon France; ^3^ LYSARC, the Lymphoma Study Academic Research Organization Lyon France; ^4^ Hematology Department University Hospital of Grenoble, Université Grenoble‐Alpes Grenoble France; ^5^ Hematology Department Saint‐Louis Hospital Paris France; ^6^ Hematology Department Bergonié Institute Bordeaux France; ^7^ Université Bourgogne Europe, CHU Dijon Bourgogne, Hématologie, INSERM1231 CTM Equipe Epi2THM Dijon France; ^8^ Hematology Department University Hospital of Strasbourg Strasbourg France; ^9^ Hematology Department Mondor Hospital Creteil France; ^10^ Hematology Department University Hospital of Bordeaux Bordeaux France; ^11^ Hematology Department University Hospital of Saint‐Étienne Saint Etienne France; ^12^ Hematology Department University Hospital of Besancon Besancon France; ^13^ Hematology Department University Hospital of Nantes Nantes France; ^14^ Hematology Department Univ Toulouse, CHU Toulouse, IUCT‐Oncopole Toulouse France; ^15^ Hematology Department Hospital of Libourne Libourne France; ^16^ Hematology Department Hospital of Vannes Vannes France; ^17^ Hematology and Cellular Department Saint Vincent Hospital, Catholic University of Lille Lille France; ^18^ Hematology Department Hospital of Dunkerque Dunkerque France; ^19^ Hematology Department University Hospital of Rennes Rennes France; ^20^ Hematology Department Hospital of Chambery Chambery France; ^21^ Hematology Department Curie Institute Saint‐Cloud France; ^22^ Hematology Department Hospital of Roubaix Roubaix France; ^23^ Hematology Department Clinique Mutualiste des Eaux Claires Grenoble France; ^24^ Hematology Department Hospital of Arras Arras France; ^25^ Hematology Department Lyon‐Sud Hospital, Hospices Civils de Lyon Pierre‐Bénite France

**Keywords:** LBCL, primary refractory and early relapse, second‐line

To the Editor,

While immunochemotherapy (IC) cures more than 50% of patients with large B‐cell lymphoma (LBCL), 30%–40% experience refractory or relapsed disease.^1^ Refractory to or experiencing progressive disease within 12 months of frontline IC (R/R12) represents a critical and discriminating prognostic factor. Before the availability of second‐line (2L) Chimerci antigen receptor (CAR) T‐cell therapy, fit patients were treated with intensive salvage IC with the intention of proceeding to autologous haematopoietic stem cell transplantation (AHSCT). Nevertheless, response rates to salvage therapy remained low (approximately 30%–40%), limiting the number of patients who ultimately underwent AHSCT, and durable long‐term remission was achieved in only a minority of patients.^2^, ^3^

TRANSFORM and ZUMA‐7 trials established CAR T cells (axi‐cel and liso‐cel) as the new 2L standard for AHSCT‐eligible R/R12 patients, with superior event‐free survival (EFS) (29.5 and 8.3 months, respectively) compared with the 2.0–3.0 months observed with salvage chemotherapy.^4^, ^5^ For AHSCT‐ineligible R/R12 patients, 2L CAR T cells approval relied on phase 2 ALYCANTE and PILOT trials (axicabtagène ciloleucel; liso [axi‐cel] and lisocabtagene maraleucel [liso‐cel]) with no comparative arms.^6^, ^7^ With the inherent selection bias of prospective interventional trials, prospective real‐world data (RWD) could help characterize R/R12 LBCL and provide complementary information to clinical trials. We analysed LBCL patients from the prospective multicentre cohort Real‐world dAta in LYmphoma patients and Survival in Adults (REALYSA) (NCT03869619)^8^ to characterize the clinical features, treatment patterns and outcomes of R/R12 patients in the pre‐2L CAR T‐cell era and to provide a benchmark for current therapeutic challenges.

## OVERALL STUDY DESIGN AND DATA COLLECTION METHODS

We analysed data of R/R12 LBCL patients who were included between November 2018 and September 2023. The REALYSA study was conducted in accordance with the Declaration of Helsinki, with ethics committee approval and data monitoring/quality control protocols described elsewhere.^9^ This period covers the landscape prior to the European approval of 2L CAR T‐cell therapy. Data were updated on 4 October 2025, ensuring mature follow‐up for the analysed cohort. All participants provided written informed consent at enrolment.

## STUDY POPULATION

Eligible patients were identified from the REALYSA cohort and met the following criteria: (i) histologically confirmed LBCL; (ii) treatment with frontline anthracycline‐ and anti‐CD20‐based IC; and (iii) occurrence of R/R12 disease, encompassing both primary refractory disease and early relapse: primary refractory disease was defined as stable disease (SD) or progressive disease (PD) at the interim or end‐of‐treatment (EOT) evaluation or a partial response (PR) at the EOT followed by progression within 12 months or complete response (CR) with relapse ≤3 months; relapse was defined as disease recurrence >3 months and within 12 months after achieving a CR, according to definition of 2L phase 3 trials.^4^, ^5^, ^10^ AHSCT eligibility was recorded as a specific Case Report Form (CRF) criterion.

## END‐POINTS OF INTEREST

The primary end‐point was EFS from the initiation of first‐line (1L) and second‐line (2L) therapy. EFS was defined as the time from the start of the respective line to disease progression, relapse, initiation of a subsequent treatment line or death from any cause, whichever comes first. Secondary end‐points included the overall response rate (ORR), CR rate and overall survival (OS), which were calculated from the initiation of 1L and 2L therapy, as well as the duration of response (DOR).

Within the REALYSA LBCL cohort (*n* = 2218), the final study population comprised 351 patients (Figure S1). Among these, 264 patients (75.2%) had primary refractory disease and 87 patients (24.8%) had relapsed, with a median of 0.9 months (range, 0–13) between the end of the first‐line response evaluation and the initiation of 2L therapy. The patient characteristics are summarized in Table 1.

**TABLE 1:** Characteristics of second‐line R/R12 patients at diagnosis and at second line.

*n* (%)	All (*n* = 351)	Primary refractory (*n* = 264)	Relapsed (*n* = 87)
Centres			
University Hospital	264 (75.2)	199 (75.4)	65 (74.7)
General Hospital	50 (14.2)	40 (15.2)	10 (11.5)
Cancer Centre	21 (6.0)	15 (5.7)	6 (6.9)
Private Clinic	16 (4.6)	10 (3.8)	6 (6.9)
Histological subtypes			
LBCL	276 (78.6)	202 (76.4)	74 (85)
GCB	98 (27.9)	69 (26.1)	29 (33.3)
Non‐GCB	111 (31.6)	83 (31.4)	28 (32.2)
NOS	67 (19.1)	50 (18.9)	17 (19.5)
HGBL	43 (12.3)	34 (12.9)	9 (2.3)
With *MYC/BCL2* and/or *BCL6* rearrangement	22 (6.3)	20 (7.6)	2 (2.3)
PMBL	14 (4.0)	13 (4.9)	1 (1.1)
Low‐grade component at diagnosis	30 (8.5)	23 (8.7)	7 (8)
At diagnosis			
Age (years, median)	68.3 (21–95)	67.6 (21–95)	69.6 (28–86)
65–75	106 (30.2%)	78 (29.5%)	28 (32.2%)
≥75	106 (30.2%)	77 (29.2%)	29 (33.3%)
Gender Male/Female	1.8	2	1.5
Ann‐Arbor stage			
I/II	46 (13.1)	38 (4.4)	8 (9.2)
III/IV	305 (86.9)	226 (85.6)	79 (90.8)
Extra‐nodal locations			
No	148 (42.2)	108 (40.9)	40 (46.0)
Yes	203 (57.8)	156 (59.1)	47 (54.0)
Elevated LDH level			
No	56 (16.0)	37 (14.0)	19 (21.8)
Yes	285 (81.2)	219 (83.0)	66 (75.9)
IPI score			
0–1	35 (10.0)	25 (9.5)	10 (11.5)
≥2	316 (90.0)	239 (90.6)	77 (88.5)
PS status			
0–1	243 (69.2)	175 (66.3)	68 (78.2)
≥2	108 (30.8)	89 (33.7)	19 (21.8)
At second line (study entry)			
Age (years, median)	68.6 (21–96)	68 (21–96)	71.1 (29–87)
65–75	111 (31.6%)	80 (30.3%)	31 (35.6%)
≥75	108 (30.8%)	78 (29.5%)	30 (34.5%)
Ann‐Arbor stage			
I/II	62 (19.6)	44 (18.4)	18 (23.4)
III/IV	254 (80.4)	195 (81.6)	59 (76.6)
Elevated LDH level			
No	115 (34.3)	85 (34)	30 (35.3)
Yes	168 (50.1)	125 (50)	43 (50.6)
IPI score			
0–1	49 (18.1)	36 (18.3)	13 (17.6)
≥2	222 (81.9)	161 (81.7)	61 (82.4)
PS status			
0–1	234 (69.6)	179 (71)	55 (65.5)
≥2	102 (30.4)	73 (29)	29 (34.5)
CNS involvement	50 (16.0)	34 (15.0)	16 (18.8)

Abbreviations: ABC, activated B‐cell; CNS, central nervous system; GC, germinal centre; HGBL, high‐grade B‐cell lymphoma (HGBL‐NOS and HGBL with *MYC* and *BCL2* and/or *BCL6* rearrangement); IPI, International Prognostic Index; LBCL, large B‐cell lymphoma; LDH, lactate dehydrogenase; NOS, not otherwise specified; PMBL, primary mediastinal B‐cell lymphoma; PS, Performance status.

At initial diagnosis, the R/R12 study population (*n* = 351) presented with high‐risk features, with an International prognotic index (IPI) score ≥2 in 90.0% of cases (*n* = 316). IC consisted of R‐CHOP (*n* = 255, 72.6%), R‐mini CHOP (*n* = 72, 20.5%) and R‐high‐dose CHOP (*n* = 24, 6.8%). SD/PD was documented for 192 patients (54.7%); 37 patients (10.5%) achieved only a PR at the EOT before progressing within 12 months. 122 patients (34.8%) achieved a CR but relapsed early: 35 (28.7%) within 3 months, 36 (29.5%) at 3–6 months and 51 (41.8%) at 6–12 months post‐EOT (Table S1). Among the R/R12 study population, the median EFS and OS from first‐line initiation were 5.5 months (95% confidence interval [CI], 5–5.9) and 27.4 months (95% CI, 22.1–35.5) respectively. Among patients in CR at the end of 1L, relapse timing was highly prognostic: the median OS (calculated from the date of progression or the date of 2L initiation) was 16.2 months (95% CI, 9.8–NR) for relapses within 3 months, 23.2 months (95% CI, 10.3–NR) at 3–6 months and 42.1 months (95% CI, 17.9–NR) at 6–12 months (NR: Not Reached).

At 2L initiation, clinical characteristics are presented in Table 1. Based on physician evaluation 28.5% of the cohort (*n* = 100/351) was considered eligible for AHSCT. Regarding 2L therapy, 50.0% of the patients (*n* = 145) received intensive regimens (e.g. R‐DHAC/DHAOX, R‐ICE), 26.9% (*n* = 78) received less intensive chemotherapy (e.g. R‐GemOx) and 9.0% (*n* = 26) received central nervous system [CNS]‐directed treatments. Despite 100 patients being initially deemed transplant‐eligible, only 25 patients (7.1% of the total cohort) ultimately underwent AHSCT due to an insufficient response to salvage chemotherapy, representing a successful transplantation rate of only 25.0% among eligible candidates.

With a median follow‐up of 28 months (range, 0.1–67.4) from 2L initiation, 274 EFS events occurred, primarily driven by progression or relapse (61.0%, *n* = 214), the other patients dead without prior event. For the entire cohort, the median EFS and OS were 2.9 months [95% CI, 2.5–3.7] and 18.8 months (95% CI, 15.9–28.0) respectively (Figure 1A,B). Among 343 evaluable patients at the end of the 2L (28 died before the EOT and 8 were not evaluated), 92 (26.8%) achieved a CR and 31 (9.8%) achieved a PR; for those in CR, the 36‐month DOR was 67.6% (Figure 1C). The outcomes were significantly worse for primary refractory patients than for those who relapsed, with a median PFS of 2.8 vs. 6.4 months (*p* = 0.02), a median EFS of 2.5 vs. 5.3 months (*p* = 0.02) and a median OS of 17.4 vs. 40.0 months (*p* = 0.07) (Figure 1D,E). The median DOR was not reached in either refractory or relapsed patients. The estimated 24‐month DOR was 77.7% (95% CI, 63.2–87.1) among refractory patients and 51.3% (95% CI, 29.0–69.8) among patients with early relapse (*p* = 0.07).

**FIGURE 1:**
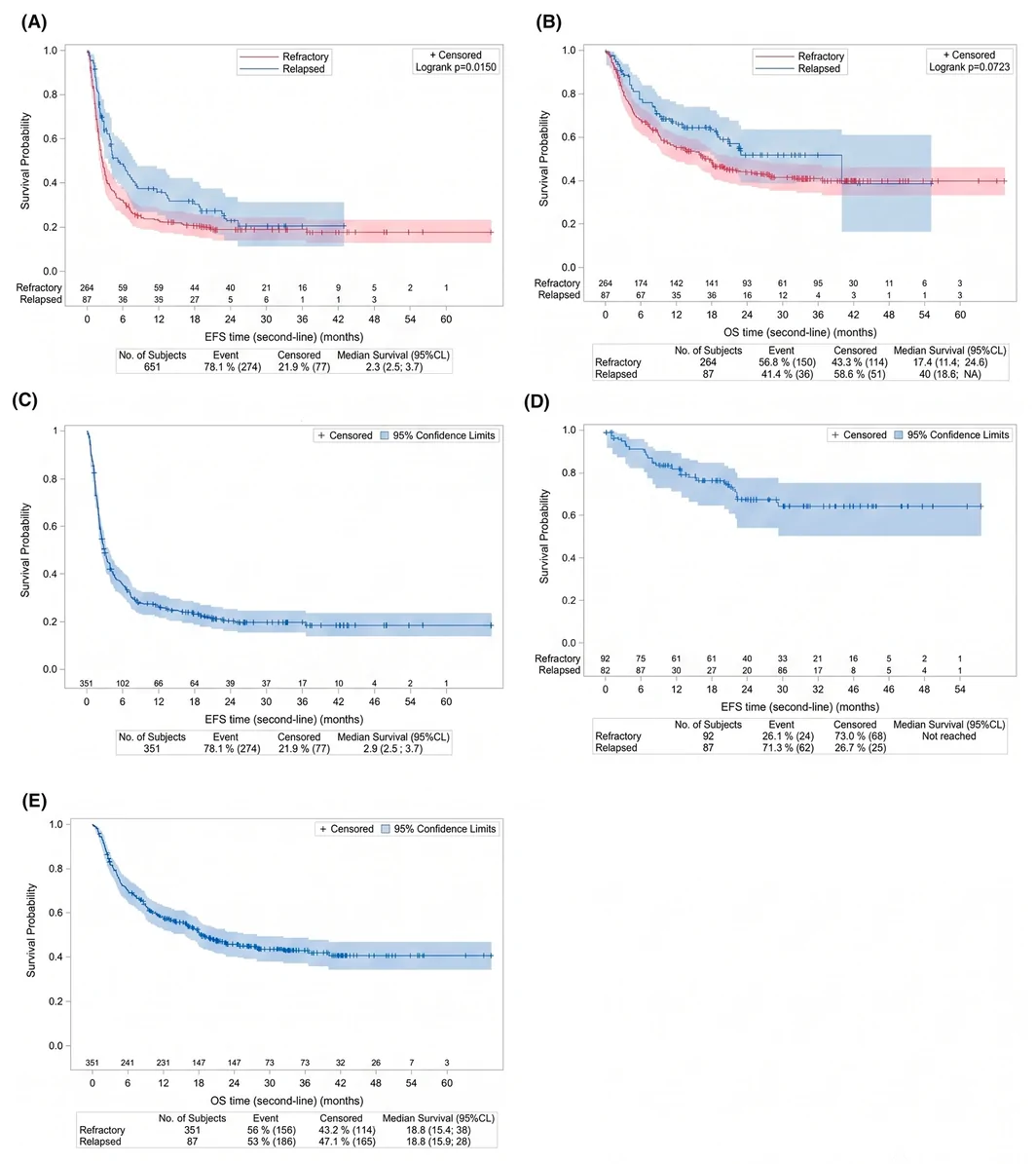
Outcomes and duration of response of patients with R/R12 in the REALYSA cohort (*n* = 351). (A) Event‐free survival after second‐line treatment for patients with R/R12. (B) Overall survival after second‐line treatment for patients in the R/R12 group. (C) Duration of complete response for patients who achieved a CR at the end of second‐line treatment. (D and E) Event‐free survival and overall survival after second‐line treatment for patients who experienced R/R12 relapse or refractory disease. DOCR, duration of complete response; EFS, event‐free survival; OS, overall survival; R/R12, patients refractory or relapsing within 12 months.

In this analysis of 351 R/R12 LBCL patients from a prospective RWD cohort treated in the pre‐2L CAR T‐cell era, we provide real‐world benchmark data complementing pivotal trials. From 2L initiation, median EFS was 2.9 months and median OS was 18.8 months.

These figures are broadly consistent with the control arms of landmark trials in both the AHSCT‐eligible and AHSCT‐ineligible settings. In the AHSCT‐eligible setting, ZUMA‐7 and TRANSFORM reported median EFS between 2.0–2.4 months and OS between 29.9–35.1 months in the standard of care (SOC) arm. In the AHSCT‐ineligible setting, the STARGLO trial reported a median EFS of 2.6 months and an OS of 12.5 months in the comparator arm, and in the DALY2‐EU trial, median EFS with R‐Gemox was 2.5 months.^11^ Despite our mixed‐eligibility population being substantially older (median age of 68 years, 31% >75 years) with more PS >1 patients (31%)—profiles that are systematically excluded from randomized controlled trials—our EFS was comparable to or exceeded the control arms of these studies, while OS fell between the AHSCT‐ineligible benchmark (STARGLO) and the AHSCT‐eligible benchmarks (ZUMA‐7 and TRANSFORM).^4^, ^5^, ^10^ In the absence of a randomized control arm, our RWD cohort may function as a synthetic control arm, providing an external reference point against which novel therapies or institutional strategies could be evaluated.

Despite a median EFS of 2.9 months, reflecting early progression on 2L therapy in a substantial proportion of patients, a median OS of 18.8 months suggests an efficacy of salvage treatments. Indeed, 34.0% of our cohort (*n* = 84/247) proceeded to CAR T‐cell therapy in 3L, highlighting the importance of early identification of eligible patients at the 2L relapse.^12^, ^13^ The survival benefit conferred by subsequent CAR T‐cell therapy in this context is further supported by data recently presented by Brisou et al.^14^ These data reinforce the interpretation that the OS observed in our cohort is not simply a reflection of the natural history of R/R LBCL after 2L failure, but is meaningfully shaped by access to subsequent CAR T‐cell therapy.

Frontline trials often rely on IPI to define high‐risk patients.^2^ Yet, as highlighted by Ghesquières et al., even within this high‐risk IPI‐defined population, outcomes remain heterogeneous. This IPI‐based stratification, however, does not fully capture the subset of R/R12 patients. R/R12 is now established in international guidelines as one of the most critical prognostic cut‐offs in LBCL.^1^, ^3^, ^12^ Strikingly, the median EFS of 2.9 months observed in our R/R12 cohort compares unfavourably even with the worst IPI strata previously reported (median EFS 5.5 months for IPI 2–5 patients).^9^ Within our R/R12 cohort, outcomes were strongly correlated with the depth and the timing of response to first‐line therapy, revealing critical heterogeneity.^15^ These data delineate a chemoresistant or early clonal selection subset most in need of next‐generation novel strategies, for whom interim PET‐CT and circulating tumour Deoxyribonucleic Acid (DNA)‐guided approaches may enable earlier identification and potentially inform the design of next‐generation frontline and salvage trials.

In conclusion, these analyses from R/R12 LBCL patients demonstrated that conventional salvage chemotherapy yields poor outcomes. These data validate the external applicability of clinical trials, reveal prognostic heterogeneity with implications for first‐line risk stratification in ongoing trials, and establish historical comparators for evaluating CAR T‐cell therapy and emerging bispecific antibody alternatives in real‐world practice.

## AUTHOR CONTRIBUTIONS

**Stéphanie Guidez:** Conceptualization; supervision; writing – original draft; resources; investigation; writing – review and editing; validation; methodology. **Bastien Lesné:** Conceptualization; methodology; software; formal analysis; writing – review and editing. **Fanny Cherblanc:** Conceptualization; methodology; writing – review and editing. **Aurélien Belot:** Conceptualization; methodology; software; data curation; formal analysis; writing – review and editing. **Alice Pauc:** Investigation; resources; writing – review and editing. **Pascale Cony‐Makhoul:** Resources; writing – review and editing. **Krimo Bouabdallah:** Resources; writing – review and editing; investigation. **Fontanet Bijou:** Resources; writing – review and editing; investigation. **Adrien Chauchet:** Resources; writing – review and editing; investigation. **Luc Mathieu Fornecker:** Resources; writing – review and editing; investigation. **Remy Gressin:** Investigation; resources; writing – review and editing. **Sandy Amorim:** Investigation; resources; writing – review and editing. **Ludovic Fouillet:** Resources; writing – review and editing; investigation. **Jehan Dupuis:** Resources; writing – review and editing; investigation. **Catherine Thieblemont:** Resources; writing – review and editing; investigation. **Sylvain Carras:** Resources; writing – review and editing; investigation. **Roch Houot:** Investigation; resources; writing – review and editing. **Sophie Dennetiere:** Investigation; resources; writing – review and editing. **Gaelle Laboure:** Investigation; resources; writing – review and editing. **Cédric Rossi:** Resources; writing – review and editing; investigation. **Carole Soussain:** Investigation; resources; writing – review and editing. **Antoine Bonnet:** Investigation; resources; writing – review and editing. **Hervé Ghesquières:** Investigation; conceptualization; resources; writing – review and editing. **Christophe Fruchart:** Investigation; resources; writing – review and editing. **Pauline Lionne‐Huyghe:** Investigation; resources; writing – review and editing. **Gian Matteo Pica:** Investigation; resources; writing – review and editing. **Cécile Leyronnas:** Investigation; resources; writing – review and editing. **Loic Ysebaert:** Resources; writing – review and editing; investigation. **Pierre Sesques:** Conceptualization; methodology; investigation; resources; writing – review and editing; writing – original draft; supervision; validation. **Benoit Tessoulin:** Resources; writing – review and editing; investigation.

## FUNDING INFORMATION

The REALYSA study is funded by several commercial organizations (Roche, Takeda, Janssen, Amgen, Celgene‐BMS, AbbVie and AstraZeneca) and sponsored by the Hospices Civils de Lyon. The present specific DLBCL analysis was funded by BMS.

## CONFLICT OF INTEREST STATEMENT

The authors declare that they have no competing interests.

## Supporting information


**Figure S1.** Study population flowchart defining the different analysis datasets.
**Table S1.** Treatment and Best response for R/R12 patients at first‐line immunochemotherapy (*n* = 351).

## Data Availability

The datasets generated during the current study are available from the corresponding author upon reasonable request. Given that the REALYSA cohort is being developed according to a specific collaboration and data‐sharing plan that encourages collaboration and the use of the resource, external collaborators may contact the corresponding author. Although use by researchers associated with the REALYSA cohort will be given priority, all requests will be considered by the Executive Committee.

## References

[1] Crump M , Neelapu SS , Farooq U , Van Den Neste E , Kuruvilla J , Westin J , et al. Outcomes in refractory diffuse large B‐cell lymphoma: results from the international SCHOLAR‐1 study. Blood. 2017;130(16):1800–1808. 10.1182/blood-2017-03-769620 PMC5649550 28774879

[2] Sehn LH , Salles G . Diffuse large B‐cell lymphoma. N Engl J Med. 2021;384(9):842–858. 10.1056/NEJMra2027612 PMC8377611 33657296

[3] Gisselbrecht C , Glass B , Mounier N , Singh Gill D , Linch DC , Trneny M , et al. Salvage regimens with autologous transplantation for relapsed large B‐cell lymphoma in the rituximab era. J Clin Oncol. 2010;28(27):4184–4190. 10.1200/JCO.2010.28.1618 PMC3664033 20660832

[4] Abramson JS , Solomon SR , Arnason J , Johnston PB , Glass B , Bachanova V , et al. Lisocabtagene maraleucel as second‐line therapy for large B‐cell lymphoma: primary analysis of the phase 3 TRANSFORM study. Blood. 2023;141(14):1675–1684. 10.1182/blood.2022018730 PMC10646768 36542826

[5] Locke FL , Miklos DB , Jacobson CA , Perales MA , Kersten MJ , Oluwole OO , et al. Axicabtagene ciloleucel as second‐line therapy for large B‐cell lymphoma. N Engl J Med. 2022;386(7):640–654. 10.1056/NEJMoa2116133 34891224

[6] Houot R , Bachy E , Cartron G , Gros FX , Morschhauser F , Oberic L , et al. Axicabtagene ciloleucel as second‐line therapy in large B cell lymphoma ineligible for autologous stem cell transplantation: a phase 2 trial. Nat Med. 2023;29(10):2593–2601. 10.1038/s41591-023-02572-5 PMC10579056 37710005

[7] Sehgal A , Hoda D , Riedell PA , Ghosh N , Hamadani M , Hildebrandt GC , et al. Lisocabtagene maraleucel as second‐line therapy in adults with relapsed or refractory large B‐cell lymphoma who were not intended for haematopoietic stem cell transplantation (PILOT): an open‐label, phase 2 study. Lancet Oncol. 2022;23(8):1066–1077. 10.1016/S1470-2045(22)00339-4 35839786

[8] Ghesquières H , Rossi C , Cherblanc F , Le Guyader‐Peyrou S , Bijou F , Sujobert P , et al. A French multicentric prospective prognostic cohort with epidemiological, clinical, biological and treatment information to improve knowledge on lymphoma patients: study protocol of the « REal world dAta in LYmphoma and survival in adults » (REALYSA) cohort. BMC Public Health. 2021;21(1):432. 10.1186/s12889-021-10433-4 PMC7927409 33653294

[9] Ghesquières H , Cherblanc F , Belot A , Micon S , Bouabdallah KK , Esnault C , et al. Challenges for quality and utilization of real‐world data for diffuse large B‐cell lymphoma in REALYSA, a LYSA cohort. Blood Adv. 2024;8(2):296–308. 10.1182/bloodadvances.2023010798 PMC10824688 37874913

[10] Abramson JS , Ku M , Hertzberg M , Huang HQ , Fox CP , Zhang H , et al. Glofitamab plus gemcitabine and oxaliplatin (GemOx) versus rituximab‐GemOx for relapsed or refractory diffuse large B‐cell lymphoma (STARGLO): a global phase 3, randomised, open‐label trial. Lancet. 2024;404(10466):1940–1954. 10.1016/S0140-6736(24)01774-4 39550172

[11] Borchmann P , Van Meerten T , Bories P , Dreger P , Gopsca L , Mutsaers P , et al. Zamtocabtagene autoleucel, a tandem CD20‐CD19 directed CAR‐T cell therapy as second‐line treatment for relapsed/refractory large B‐cell lymphoma: primary analysis of the randomized, pivotal DALY 2‐EU study. Blood. 2025;146(Supplement 1):669. 10.1182/blood-2025-669

[12] Sesques P , Manson G , Dubois S , Gros FX , Brisou G , Sarkozy C , et al. Large B‐cell lymphoma: the LYSA pragmatic guidelines. Eur J Cancer. 2026;232:116070. 10.1016/j.ejca.2025.116070 41352004

[13] Thieblemont C , Gomes Da Silva M , Leppä S , Lenz G , Cottereau AS , Fox C , et al. Large B‐cell lymphoma (LBCL): EHA clinical practice guidelines for diagnosis, treatment, and follow‐up. HemaSphere. 2025;9(9):e70207. 10.1002/hem3.70207 PMC12456099 40995463

[14] Brisou G , Belot A , Bachy E , Ghesquieres H , Gros FX , Bouabdallah K , et al. Comparison of axicabtagene ciloleucel to standard regimens as second line treatment for large B cell lymphoma in real life: a lysa study from descar‐T and realysa registries. Blood. 2025;146(Supplement 1):2764. 10.1182/blood-2025-2764

[15] Bock AM , Mwangi R , Wang Y , Khurana A , Maurer MJ , Ayers A , et al. Defining primary refractory large B‐cell lymphoma. Blood Adv. 2024;8(13):3402–3415. 10.1182/bloodadvances.2024012760 PMC11255370 38669353

