# Supplementary material for: Outcomes of refractory and early‐relapsed large B‐cell lymphoma patients after first‐line treatment in the prospective Real‐world dAta in LYmphoma patients and Survival in Adults (REALYSA) cohort in the pre‐CAR T era
Source: Br J Haematol. 2026 Aug 30;209(5):2295–301. doi: 10.1111/bjh.70812 (PMC13748374; doi:10.1111/bjh.70812)
Supplement: Supplementary file 1 [file BJH-209-2295-s001.docx]

**Supplemental materials.**

**Supplemental Figure 1: Study population flowchart defining the different analysis datasets.**

REALYSA DLBCL patients enrolled & included before the cut-off date

(N=2218)

DLBCL patients treated with antiCD20 & anthracycline for their 1st line (N=2133)

Patients not treated during their 1st line or with a treatment that does not include a combination of antiCD20 + anthracycline (N=85)

Patients in relapse and treated in a 2nd line (popT, N=465)

DLBCL patients treated with antiCD20 and treated in a 2nd line and with R/R within 12 months and excluding 2nd line interventional trials (popT_NIT_RR12, N=351)

**Supplemental Table 1**: **Treatment and Best response for R/R12 patients at first line immunochemotherapy(n=351).**

| **n (%)** | **All**  **(n=351)** | **Primary refractory**  **(n=264)** | **Relapsed**  **(n=87)** |
| --- | --- | --- | --- |
| **First line** | | | |
| **Treatment in first line**  **R-CHOP**  **R-mini CHOP**  **R-high-dose CHOP** | 255 (72.6)  72 (20.5)  24 (6.8) | 192 (72.7)  52 (19.7)  20 (7.6) | 63 (72.4)  20 (23.0)  4 (4.6) |
| **Response at end of Line 1**  **CR**  **PR**  **SD**  **PD** | 169 (48.1)  98 (27.9)  18 (5.1)  66 (18.8) | 82 (31.1)  98 (37.1)  18 (6.8)  66 (25) | 87 (100)  0  0  0 |

**Abbreviations:** CR: complete response; PD: progressive disease; PR: partial response; SD: stable disease.

**R-high-dose CHOP correspond to R-ACVBP and R-COPADEM.**
